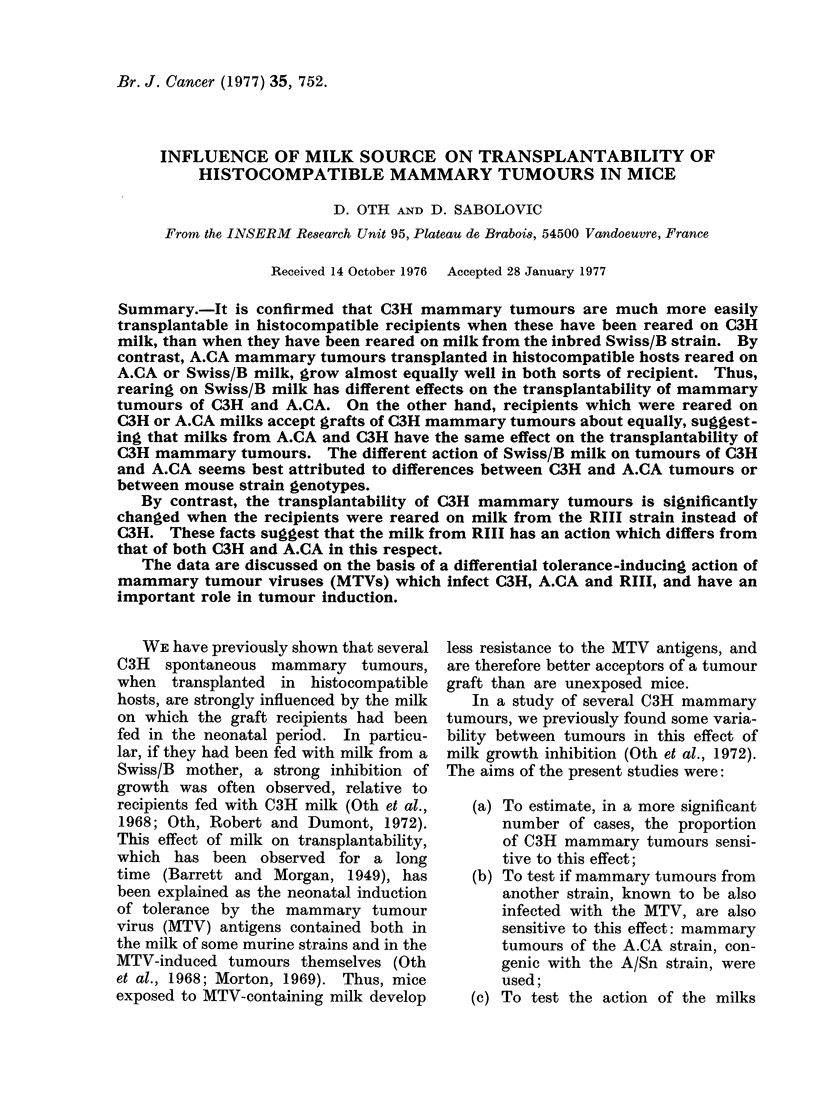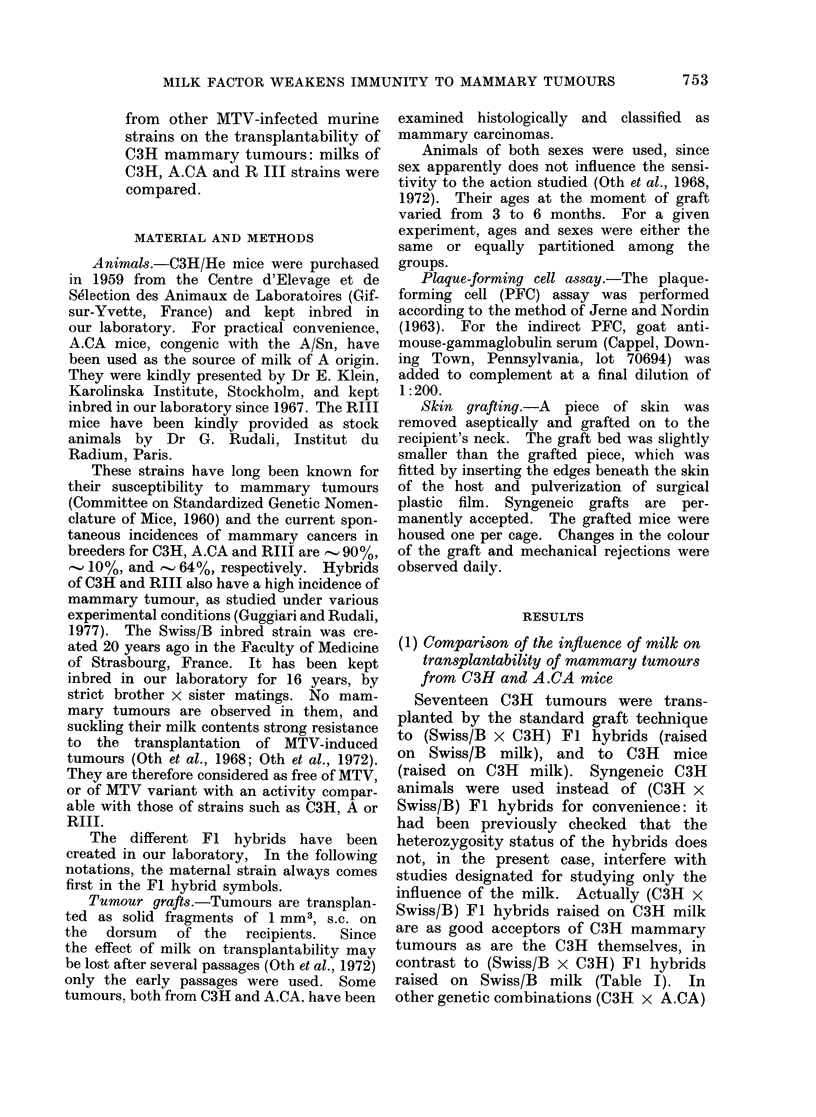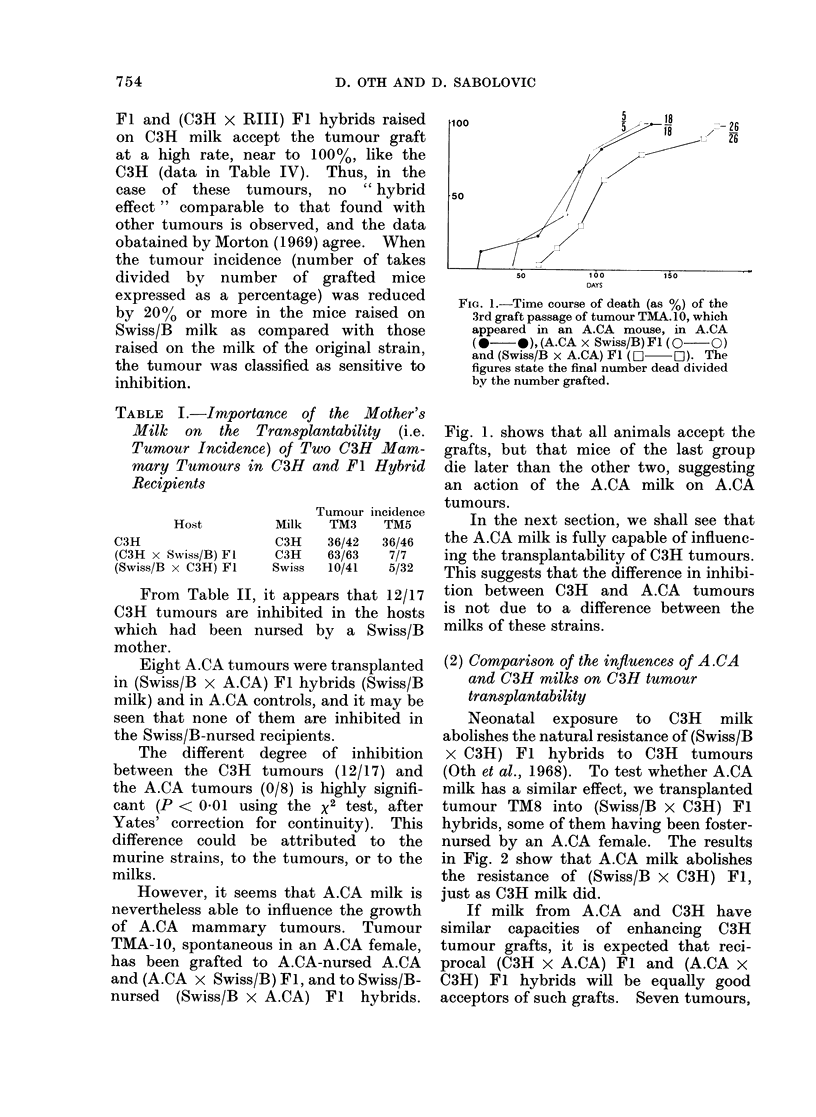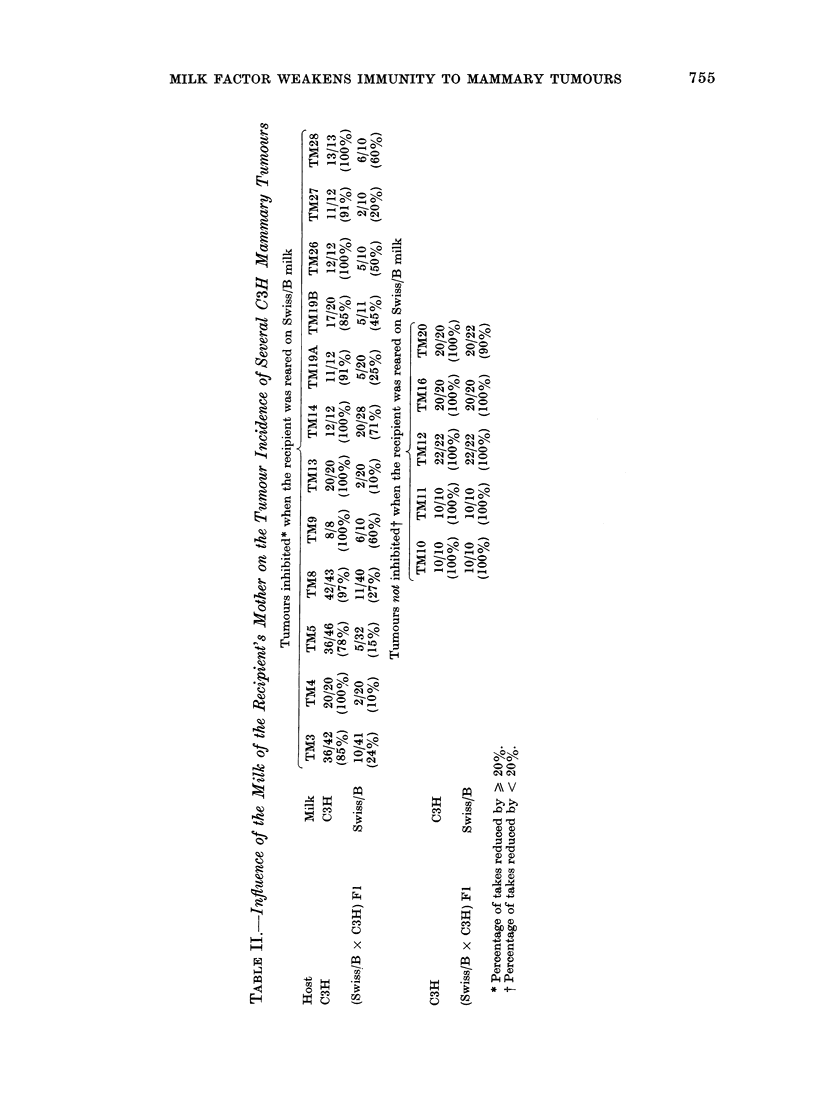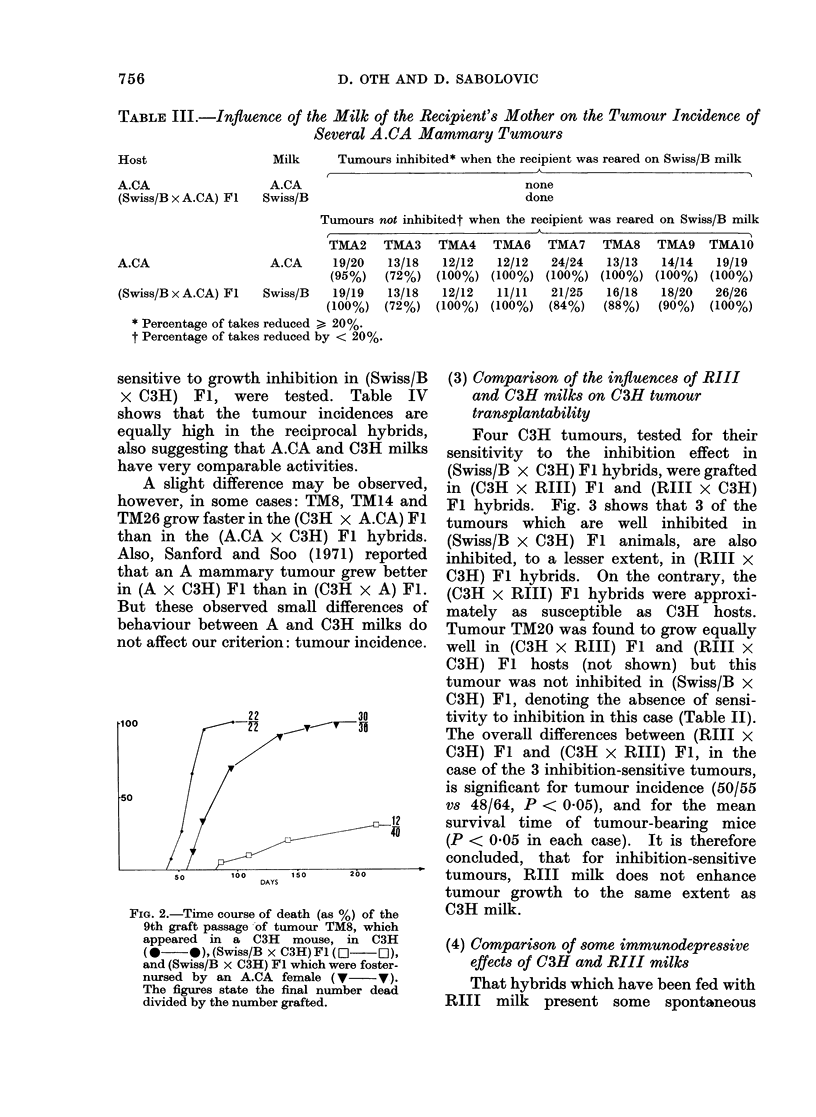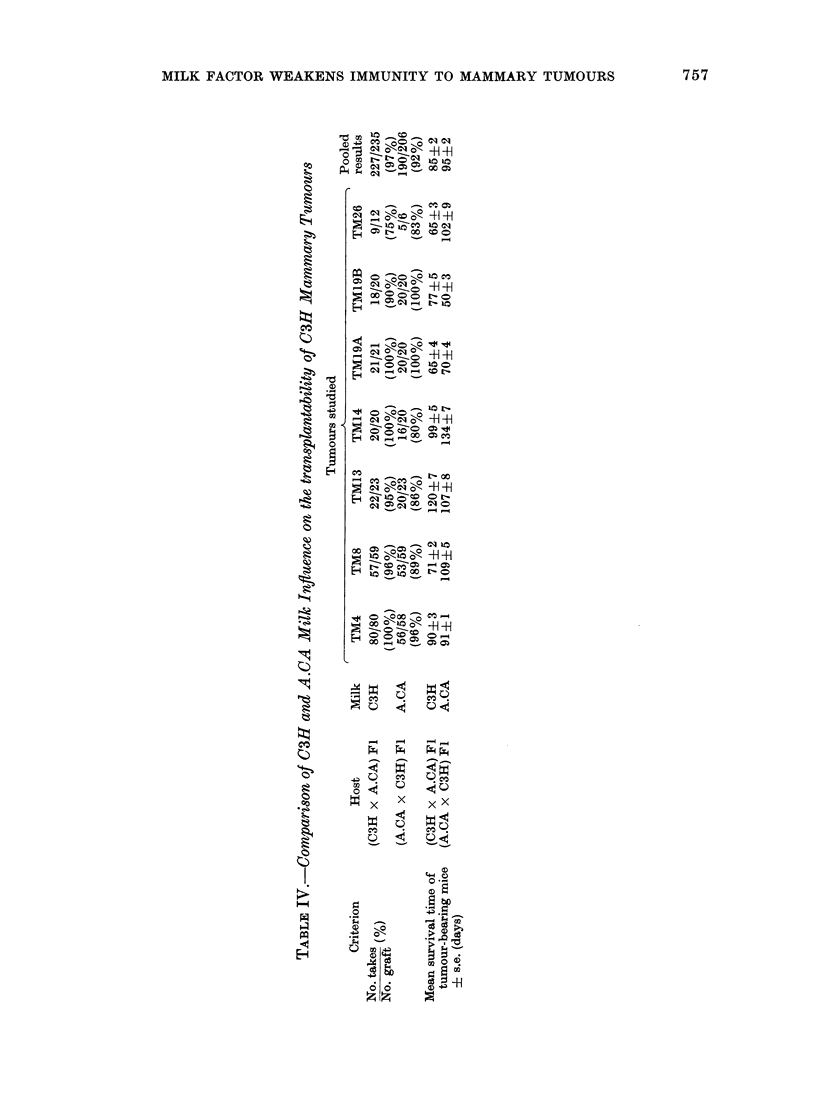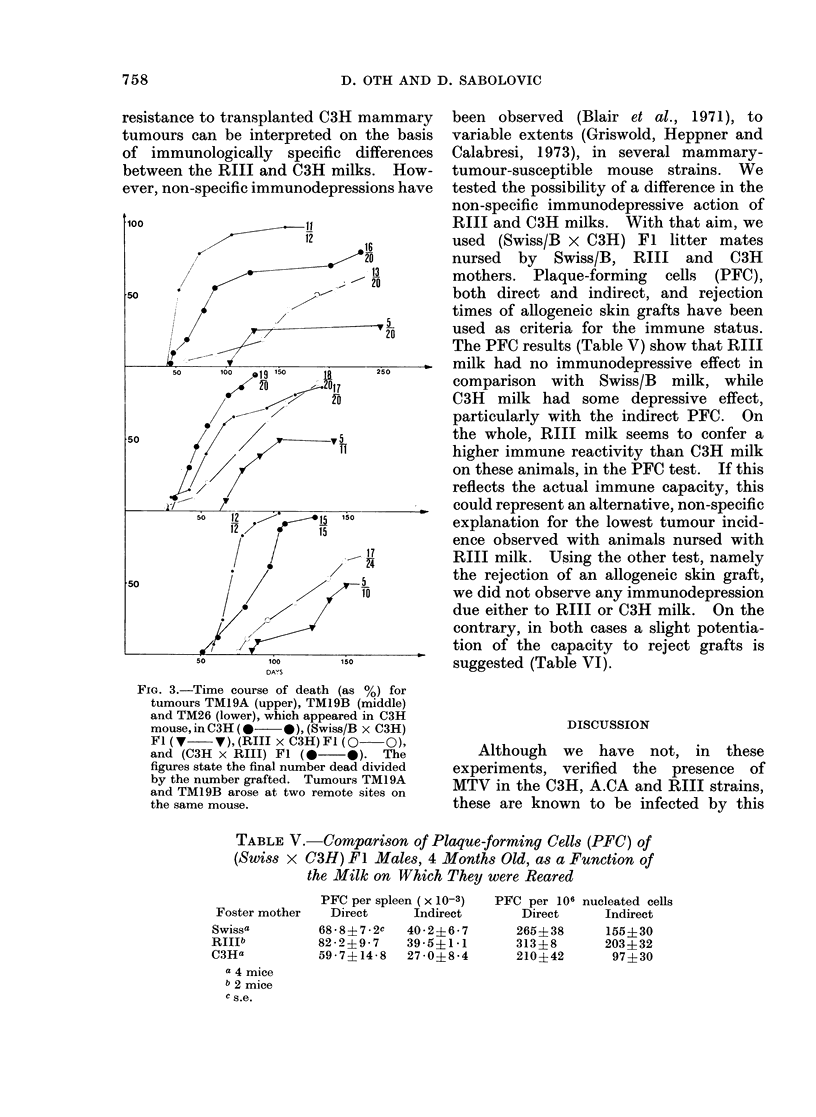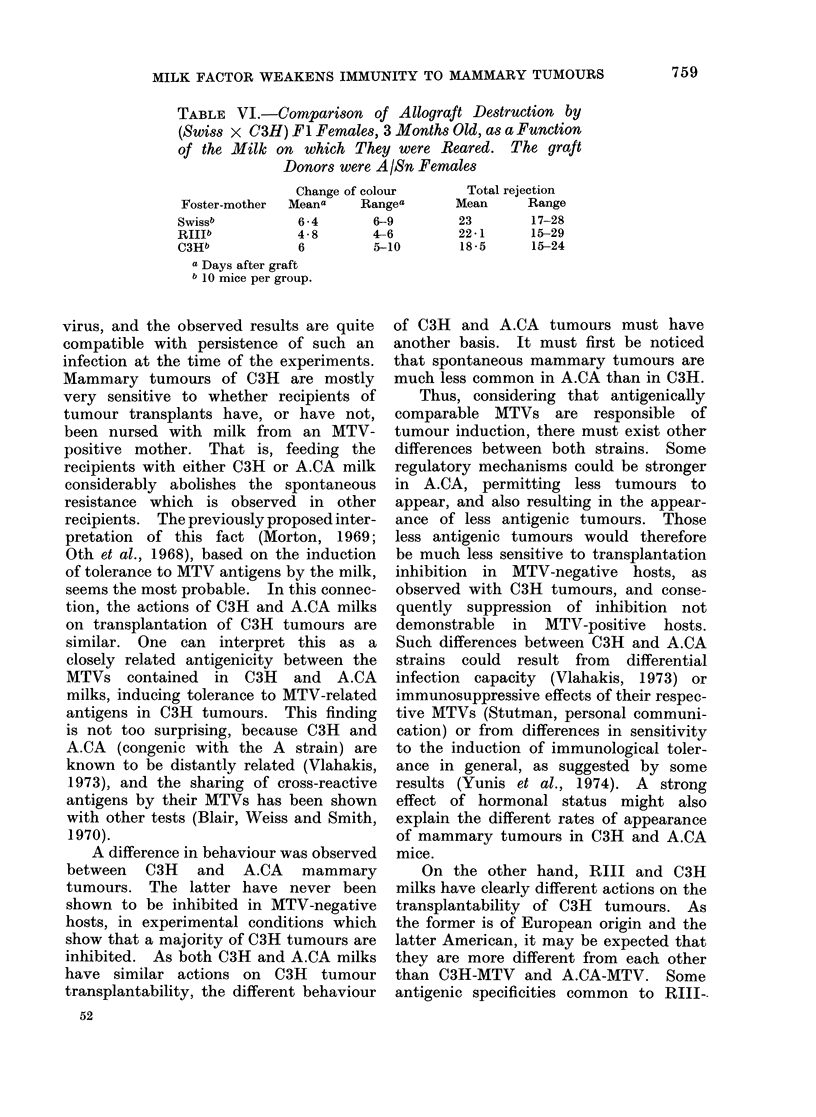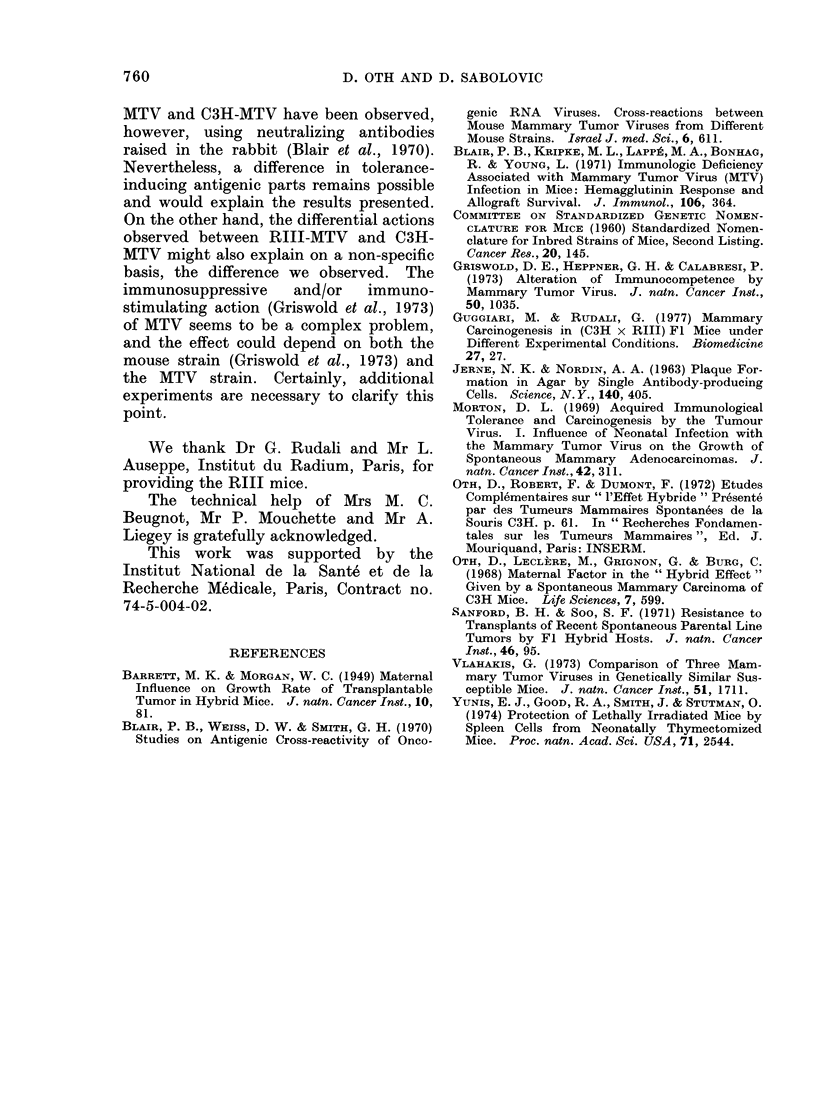# Influence of milk source on transplantability of histocompatible mammary tumours in mice.

**DOI:** 10.1038/bjc.1977.116

**Published:** 1977-06

**Authors:** D. Oth, D. Sabolovic

## Abstract

It is confirmed that C3H mammary tumours are much more easily transplantable in histocompatible recipients when these have been reared on C3H milk, than when they have been reared on milk from the inbred Swiss/B strain. By contrast, A.CA mammary tumours transplanted in histocompatible hosts reared on A.CA or Swiss/B milk, grow almost equally well in both sorts of recipient. Thus, rearing on Swiss/B milk has different effects on the transplantability of mammary tumours of C3H and A.CA. On the other hand, recipients which were reared on C3H or A.CA milks accept grafts of C3H mammary tumours about equally, suggesting that milks from A.CA and C3H have the same effect on the transplantability of C3H mammary tumours. The different action of Swiss/B milk on tumours of C3H and A.CA seems best attributed to differences between C3H and A.CA tumours or between mouse strain genotypes. By contrast, the transplantability of C3H mammary tumours is significantly changed when the recipients were reared on milk from the RIII strain instead of C3H. These facts suggest that the milk from RIII has an action which differs from that of both C3H and A.CA in this respect. The data are discussed on the basis of a differential tollerance-inducing action of mammary tumour viruses (MTVs) which infect C3H, A.CA and RIII, and have an important role in tumour induction.


*Br. J. Cancer* (1977) **35**, 752.

### INFLUENCE OF MILK SOURCE ON TRANSPLANTABILITY OF HISTOCOMPATIBLE MAMMARY TUMOURS IN MICE

D. OTH AND D. SABOLOVIC

*From the INSERM Research Unit* 95, *Plateau de Brabois*, 54500 *Vandoeuvre, France*

Received 14 October 1976 Accepted 28 January 1977

**Summary.—It is confirmed that C3H mammary tumours are much more easily transplantable in histocompatible recipients when these have been reared on C3H milk, than when they have been reared on milk from the inbred Swiss/B strain. By contrast, A.CA mammary tumours transplanted in histocompatible hosts reared on A.CA or Swiss/B milk, grow almost equally well in both sorts of recipient. Thus, rearing on Swiss/B milk has different effects on the transplantability of mammary tumours of C3H and A.CA. On the other hand, recipients which were reared on C3H or A.CA milks accept grafts of C3H mammary tumours about equally, suggesting that milks from A.CA and C3H have the same effect on the transplantability of C3H mammary tumours. The different action of Swiss/B milk on tumours of C3H and A.CA seems best attributed to differences between C3H and A.CA tumours or between mouse strain genotypes.**

**By contrast, the transplantability of C3H mammary tumours is significantly changed when the recipients were reared on milk from the RIII strain instead of C3H. These facts suggest that the milk from RIII has an action which differs from that of both C3H and A.CA in this respect.**

**The data are discussed on the basis of a differential tolerance-inducing action of mammary tumour viruses (MTVs) which infect C3H, A.CA and RIII, and have an important role in tumour induction.**

WE have previously shown that several C3H spontaneous mammary tumours, when transplanted in histocompatible hosts, are strongly influenced by the milk on which the graft recipients had been fed in the neonatal period. In particular, if they had been fed with milk from a Swiss/B mother, a strong inhibition of growth was often observed, relative to recipients fed with C3H milk (Oth *et al.*, 1968; Oth, Robert and Dumont, 1972). This effect of milk on transplantability, which has been observed for a long time (Barrett and Morgan, 1949), has been explained as the neonatal induction of tolerance by the mammary tumour virus (MTV) antigens contained both in the milk of some murine strains and in the MTV-induced tumours themselves (Oth *et al.*, 1968; Morton, 1969). Thus, mice exposed to MTV-containing milk develop less resistance to the MTV antigens, and are therefore better acceptors of a tumour graft than are unexposed mice.

In a study of several C3H mammary tumours, we previously found some variability between tumours in this effect of milk growth inhibition (Oth *et al.*, 1972). The aims of the present studies were:

(a) To estimate, in a more significant number of cases, the proportion of C3H mammary tumours sensitive to this effect;
(b) To test if mammary tumours from another strain, known to be also infected with the MTV, are also sensitive to this effect: mammary tumours of the A.CA strain, congenic with the A/Sn strain, were used;
(c) To test the action of the milks from other MTV-infected murine strains on the transplantability of C3H mammary tumours: milks of C3H, A.CA and R III strains were compared.

### MATERIAL AND METHODS

*Animals.*—C3H/He mice were purchased in 1959 from the Centre d'Elevage et de Sélection des Animaux de Laboratoires (Gif-sur-Yvette, France) and kept inbred in our laboratory. For practical convenience, A.CA mice, congenic with the A/Sn, have been used as the source of milk of A origin. They were kindly presented by Dr E. Klein, Karolinska Institute, Stockholm, and kept inbred in our laboratory since 1967. The RIII mice have been kindly provided as stock animals by Dr G. Rudali, Institut du Radium, Paris.

These strains have long been known for their susceptibility to mammary tumours (Committee on Standardized Genetic Nomenclature of Mice, 1960) and the current spontaneous incidences of mammary cancers in breeders for C3H, A.CA and RIII are $\sim 90\%$, $\sim 10\%$, and $\sim 64\%$, respectively. Hybrids of C3H and RIII also have a high incidence of mammary tumour, as studied under various experimental conditions (Guggiari and Rudali, 1977). The Swiss/B inbred strain was created 20 years ago in the Faculty of Medicine of Strasbourg, France. It has been kept inbred in our laboratory for 16 years, by strict brother $\times$ sister matings. No mammary tumours are observed in them, and suckling their milk contents strong resistance to the transplantation of MTV-induced tumours (Oth *et al.*, 1968; Oth *et al.*, 1972). They are therefore considered as free of MTV, or of MTV variant with an activity comparable with those of strains such as C3H, A or RIII.

The different F1 hybrids have been created in our laboratory, In the following notations, the maternal strain always comes first in the F1 hybrid symbols.

*Tumour grafts.*—Tumours are transplanted as solid fragments of 1 $mm^3$, s.c. on the dorsum of the recipients. Since the effect of milk on transplantability may be lost after several passages (Oth *et al.*, 1972) only the early passages were used. Some tumours, both from C3H and A.CA, have been examined histologically and classified as mammary carcinomas.

Animals of both sexes were used, since sex apparently does not influence the sensitivity to the action studied (Oth *et al.*, 1968, 1972). Their ages at the moment of graft varied from 3 to 6 months. For a given experiment, ages and sexes were either the same or equally partitioned among the groups.

*Plaque-forming cell assay.*—The plaque-forming cell (PFC) assay was performed according to the method of Jerne and Nordin (1963). For the indirect PFC, goat anti-mouse-gammaglobulin serum (Cappel, Downing Town, Pennsylvania, lot 70694) was added to complement at a final dilution of 1:200.

*Skin grafting.*—A piece of skin was removed aseptically and grafted on to the recipient's neck. The graft bed was slightly smaller than the grafted piece, which was fitted by inserting the edges beneath the skin of the host and pulverization of surgical plastic film. Syngeneic grafts are permanently accepted. The grafted mice were housed one per cage. Changes in the colour of the graft and mechanical rejections were observed daily.

### RESULTS

### (1) *Comparison of the influence of milk on transplantability of mammary tumours from C3H and A.CA mice*

Seventeen C3H tumours were transplanted by the standard graft technique to (Swiss/B $\times$ C3H) F1 hybrids (raised on Swiss/B milk), and to C3H mice (raised on C3H milk). Syngeneic C3H animals were used instead of (C3H $\times$ Swiss/B) F1 hybrids for convenience: it had been previously checked that the heterozygosity status of the hybrids does not, in the present case, interfere with studies designated for studying only the influence of the milk. Actually (C3H $\times$ Swiss/B) F1 hybrids raised on C3H milk are as good acceptors of C3H mammary tumours as are the C3H themselves, in contrast to (Swiss/B $\times$ C3H) F1 hybrids raised on Swiss/B milk (Table I). In other genetic combinations (C3H $\times$ A.CA)

F1 and (C3H × RIII) F1 hybrids raised on C3H milk accept the tumour graft at a high rate, near to 100%, like the C3H (data in Table IV). Thus, in the case of these tumours, no "hybrid effect" comparable to that found with other tumours is observed, and the data obatained by Morton (1969) agree. When the tumour incidence (number of takes divided by number of grafted mice expressed as a percentage) was reduced by 20% or more in the mice raised on Swiss/B milk as compared with those raised on the milk of the original strain, the tumour was classified as sensitive to inhibition.

TABLE I.—*Importance of the Mother's Milk on the Transplantability* (i.e. *Tumour Incidence*) *of Two C3H Mammary Tumours in C3H and F1 Hybrid Recipients*

| Host | Milk | Tumour incidence TM3 | TM5 |
|---|---|---|---|
| C3H | C3H | 36/42 | 36/46 |
| (C3H × Swiss/B) F1 | C3H | 63/63 | 7/7 |
| (Swiss/B × C3H) F1 | Swiss | 10/41 | 5/32 |

From Table II, it appears that 12/17 C3H tumours are inhibited in the hosts which had been nursed by a Swiss/B mother.

Eight A.CA tumours were transplanted in (Swiss/B × A.CA) F1 hybrids (Swiss/B milk) and in A.CA controls, and it may be seen that none of them are inhibited in the Swiss/B-nursed recipients.

The different degree of inhibition between the C3H tumours (12/17) and the A.CA tumours (0/8) is highly significant ($P < 0{\cdot}01$ using the $\chi^2$ test, after Yates' correction for continuity). This difference could be attributed to the murine strains, to the tumours, or to the milks.

However, it seems that A.CA milk is nevertheless able to influence the growth of A.CA mammary tumours. Tumour TMA-10, spontaneous in an A.CA female, has been grafted to A.CA-nursed A.CA and (A.CA × Swiss/B) F1, and to Swiss/B-nursed (Swiss/B × A.CA) F1 hybrids.

FIG. 1.—Time course of death (as %) of the 3rd graft passage of tumour TMA.10, which appeared in an A.CA mouse, in A.CA (●——●), (A.CA × Swiss/B) F1 (○——○) and (Swiss/B × A.CA) F1 (□——□). The figures state the final number dead divided by the number grafted.

Fig. 1. shows that all animals accept the grafts, but that mice of the last group die later than the other two, suggesting an action of the A.CA milk on A.CA tumours.

In the next section, we shall see that the A.CA milk is fully capable of influencing the transplantability of C3H tumours. This suggests that the difference in inhibition between C3H and A.CA tumours is not due to a difference between the milks of these strains.

(2) *Comparison of the influences of A.CA and C3H milks on C3H tumour transplantability*

Neonatal exposure to C3H milk abolishes the natural resistance of (Swiss/B × C3H) F1 hybrids to C3H tumours (Oth *et al.*, 1968). To test whether A.CA milk has a similar effect, we transplanted tumour TM8 into (Swiss/B × C3H) F1 hybrids, some of them having been foster-nursed by an A.CA female. The results in Fig. 2 show that A.CA milk abolishes the resistance of (Swiss/B × C3H) F1, just as C3H milk did.

If milk from A.CA and C3H have similar capacities of enhancing C3H tumour grafts, it is expected that reciprocal (C3H × A.CA) F1 and (A.CA × C3H) F1 hybrids will be equally good acceptors of such grafts. Seven tumours,

TABLE II.—*Influence of the Milk of the Recipient's Mother on the Tumour Incidence of Several C3H Mammary Tumours*

Tumours inhibited* when the recipient was reared on Swiss/B milk

| Host | Milk | TM3 | TM4 | TM5 | TM8 | TM9 | TM13 | TM14 | TM19A | TM19B | TM26 | TM27 | TM28 |
|---|---|---|---|---|---|---|---|---|---|---|---|---|---|
| C3H | C3H | 36/42 (85%) | 20/20 (100%) | 36/46 (78%) | 42/43 (97%) | 8/8 (100%) | 20/20 (100%) | 12/12 (100%) | 11/12 (91%) | 17/20 (85%) | 12/12 (100%) | 11/12 (91%) | 13/13 (100%) |
| (Swiss/B × C3H) F1 | Swiss/B | 10/41 (24%) | 2/20 (10%) | 5/32 (15%) | 11/40 (27%) | 6/10 (60%) | 2/20 (10%) | 20/28 (71%) | 5/20 (25%) | 5/11 (45%) | 5/10 (50%) | 2/10 (20%) | 6/10 (60%) |

Tumours *not* inhibited† when the recipient was reared on Swiss/B milk

| Host | Milk | TM10 | TM11 | TM12 | TM16 | TM20 |
|---|---|---|---|---|---|---|
| C3H | C3H | 10/10 (100%) | 10/10 (100%) | 22/22 (100%) | 20/20 (100%) | 20/20 (100%) |
| (Swiss/B × C3H) F1 | Swiss/B | 10/10 (100%) | 10/10 (100%) | 22/22 (100%) | 20/20 (100%) | 20/22 (90%) |

* Percentage of takes reduced by $\geqslant$ 20%.
† Percentage of takes reduced by $<$ 20%.

TABLE III.—*Influence of the Milk of the Recipient's Mother on the Tumour Incidence of Several A.CA Mammary Tumours*

| Host | Milk | Tumours inhibited* when the recipient was reared on Swiss/B milk |
| --- | --- | --- |
| A.CA | A.CA | none |
| (Swiss/B × A.CA) F1 | Swiss/B | done |

| Host | Milk | Tumours *not* inhibited† when the recipient was reared on Swiss/B milk | | | | | | | |
| --- | --- | --- | --- | --- | --- | --- | --- | --- | --- |
| | | TMA2 | TMA3 | TMA4 | TMA6 | TMA7 | TMA8 | TMA9 | TMA10 |
| A.CA | A.CA | 19/20 (95%) | 13/18 (72%) | 12/12 (100%) | 12/12 (100%) | 24/24 (100%) | 13/13 (100%) | 14/14 (100%) | 19/19 (100%) |
| (Swiss/B × A.CA) F1 | Swiss/B | 19/19 (100%) | 13/18 (72%) | 12/12 (100%) | 11/11 (100%) | 21/25 (84%) | 16/18 (88%) | 18/20 (90%) | 26/26 (100%) |

* Percentage of takes reduced ⩾ 20%.
† Percentage of takes reduced by < 20%.

sensitive to growth inhibition in (Swiss/B × C3H) F1, were tested. Table IV shows that the tumour incidences are equally high in the reciprocal hybrids, also suggesting that A.CA and C3H milks have very comparable activities.

A slight difference may be observed, however, in some cases: TM8, TM14 and TM26 grow faster in the (C3H × A.CA) F1 than in the (A.CA × C3H) F1 hybrids. Also, Sanford and Soo (1971) reported that an A mammary tumour grew better in (A × C3H) F1 than in (C3H × A) F1. But these observed small differences of behaviour between A and C3H milks do not affect our criterion: tumour incidence.

FIG. 2.—Time course of death (as %) of the 9th graft passage of tumour TM8, which appeared in a C3H mouse, in C3H (●——●), (Swiss/B × C3H) F1 (□——□), and (Swiss/B × C3H) F1 which were foster-nursed by an A.CA female (▼——▼). The figures state the final number dead divided by the number grafted.

(3) *Comparison of the influences of RIII and C3H milks on C3H tumour transplantability*

Four C3H tumours, tested for their sensitivity to the inhibition effect in (Swiss/B × C3H) F1 hybrids, were grafted in (C3H × RIII) F1 and (RIII × C3H) F1 hybrids. Fig. 3 shows that 3 of the tumours which are well inhibited in (Swiss/B × C3H) F1 animals, are also inhibited, to a lesser extent, in (RIII × C3H) F1 hybrids. On the contrary, the (C3H × RIII) F1 hybrids were approximately as susceptible as C3H hosts. Tumour TM20 was found to grow equally well in (C3H × RIII) F1 and (RIII × C3H) F1 hosts (not shown) but this tumour was not inhibited in (Swiss/B × C3H) F1, denoting the absence of sensitivity to inhibition in this case (Table II). The overall differences between (RIII × C3H) F1 and (C3H × RIII) F1, in the case of the 3 inhibition-sensitive tumours, is significant for tumour incidence (50/55 *vs* 48/64, $P < 0{\cdot}05$), and for the mean survival time of tumour-bearing mice ($P < 0{\cdot}05$ in each case). It is therefore concluded, that for inhibition-sensitive tumours, RIII milk does not enhance tumour growth to the same extent as C3H milk.

(4) *Comparison of some immunodepressive effects of C3H and RIII milks*

That hybrids which have been fed with RIII milk present some spontaneous

TABLE IV.—*Comparison of C3H and A.CA Milk Influence on the transplantability of C3H Mammary Tumours*

| Criterion | Host | Milk | Tumours studied: TM4 | TM8 | TM13 | TM14 | TM19A | TM19B | TM26 | Pooled results |
|---|---|---|---|---|---|---|---|---|---|---|
| $\frac{\text{No. takes}}{\text{No. graft}}$ (%) | (C3H × A.CA) F1 | C3H | 80/80 (100%) | 57/59 (96%) | 22/23 (95%) | 20/20 (100%) | 21/21 (100%) | 18/20 (90%) | 9/12 (75%) | 227/235 (97%) |
| | (A.CA × C3H) F1 | A.CA | 56/58 (96%) | 53/59 (89%) | 20/23 (86%) | 16/20 (80%) | 20/20 (100%) | 20/20 (100%) | 5/6 (83%) | 190/206 (92%) |
| Mean survival time of tumour-bearing mice ± s.e. (days) | (C3H × A.CA) F1 | C3H | 90±3 | 71±2 | 120±7 | 99±5 | 65±4 | 77±5 | 65±3 | 85±2 |
| | (A.CA × C3H) F1 | A.CA | 91±1 | 109±5 | 107±8 | 134±7 | 70±4 | 50±3 | 102±9 | 95±2 |

resistance to transplanted C3H mammary tumours can be interpreted on the basis of immunologically specific differences between the RIII and C3H milks. However, non-specific immunodepressions have been observed (Blair *et al.*, 1971), to variable extents (Griswold, Heppner and Calabresi, 1973), in several mammary-tumour-susceptible mouse strains. We tested the possibility of a difference in the non-specific immunodepressive action of RIII and C3H milks. With that aim, we used (Swiss/B × C3H) F1 litter mates nursed by Swiss/B, RIII and C3H mothers. Plaque-forming cells (PFC), both direct and indirect, and rejection times of allogeneic skin grafts have been used as criteria for the immune status. The PFC results (Table V) show that RIII milk had no immunodepressive effect in comparison with Swiss/B milk, while C3H milk had some depressive effect, particularly with the indirect PFC. On the whole, RIII milk seems to confer a higher immune reactivity than C3H milk on these animals, in the PFC test. If this reflects the actual immune capacity, this could represent an alternative, non-specific explanation for the lowest tumour incidence observed with animals nursed with RIII milk. Using the other test, namely the rejection of an allogeneic skin graft, we did not observe any immunodepression due either to RIII or C3H milk. On the contrary, in both cases a slight potentiation of the capacity to reject grafts is suggested (Table VI).

FIG. 3.—Time course of death (as %) for tumours TM19A (upper), TM19B (middle) and TM26 (lower), which appeared in C3H mouse, in C3H (●——●), (Swiss/B × C3H) F1 (▼——▼), (RIII × C3H) F1 (○——○), and (C3H × RIII) F1 (●——●). The figures state the final number dead divided by the number grafted. Tumours TM19A and TM19B arose at two remote sites on the same mouse.

DISCUSSION

Although we have not, in these experiments, verified the presence of MTV in the C3H, A.CA and RIII strains, these are known to be infected by this

TABLE V.—*Comparison of Plaque-forming Cells (PFC) of (Swiss × C3H) F1 Males, 4 Months Old, as a Function of the Milk on Which They were Reared*

| | PFC per spleen ($\times 10^{-3}$) | | PFC per $10^6$ nucleated cells | |
|---|---|---|---|---|
| Foster mother | Direct | Indirect | Direct | Indirect |
| Swiss[a] | 68·8±7·2[c] | 40·2±6·7 | 265±38 | 155±30 |
| RIII[b] | 82·2±9·7 | 39·5±1·1 | 313±8 | 203±32 |
| C3H[a] | 59·7±14·8 | 27·0±8·4 | 210±42 | 97±30 |

[a] 4 mice
[b] 2 mice
[c] s.e.

TABLE VI.—*Comparison of Allograft Destruction by (Swiss × C3H) F1 Females, 3 Months Old, as a Function of the Milk on which They were Reared. The graft Donors were A/Sn Females*

| Foster-mother | Change of colour Mean[a] | Change of colour Range[a] | Total rejection Mean | Total rejection Range |
|---|---|---|---|---|
| Swiss[b] | 6·4 | 6–9 | 23 | 17–28 |
| RIII[b] | 4·8 | 4–6 | 22·1 | 15–29 |
| C3H[b] | 6 | 5–10 | 18·5 | 15–24 |

[a] Days after graft
[b] 10 mice per group.

virus, and the observed results are quite compatible with persistence of such an infection at the time of the experiments. Mammary tumours of C3H are mostly very sensitive to whether recipients of tumour transplants have, or have not, been nursed with milk from an MTV-positive mother. That is, feeding the recipients with either C3H or A.CA milk considerably abolishes the spontaneous resistance which is observed in other recipients. The previously proposed interpretation of this fact (Morton, 1969; Oth *et al.*, 1968), based on the induction of tolerance to MTV antigens by the milk, seems the most probable. In this connection, the actions of C3H and A.CA milks on transplantation of C3H tumours are similar. One can interpret this as a closely related antigenicity between the MTVs contained in C3H and A.CA milks, inducing tolerance to MTV-related antigens in C3H tumours. This finding is not too surprising, because C3H and A.CA (congenic with the A strain) are known to be distantly related (Vlahakis, 1973), and the sharing of cross-reactive antigens by their MTVs has been shown with other tests (Blair, Weiss and Smith, 1970).

A difference in behaviour was observed between C3H and A.CA mammary tumours. The latter have never been shown to be inhibited in MTV-negative hosts, in experimental conditions which show that a majority of C3H tumours are inhibited. As both C3H and A.CA milks have similar actions on C3H tumour transplantability, the different behaviour of C3H and A.CA tumours must have another basis. It must first be noticed that spontaneous mammary tumours are much less common in A.CA than in C3H.

Thus, considering that antigenically comparable MTVs are responsible of tumour induction, there must exist other differences between both strains. Some regulatory mechanisms could be stronger in A.CA, permitting less tumours to appear, and also resulting in the appearance of less antigenic tumours. Those less antigenic tumours would therefore be much less sensitive to transplantation inhibition in MTV-negative hosts, as observed with C3H tumours, and consequently suppression of inhibition not demonstrable in MTV-positive hosts. Such differences between C3H and A.CA strains could result from differential infection capacity (Vlahakis, 1973) or immunosuppressive effects of their respective MTVs (Stutman, personal communication) or from differences in sensitivity to the induction of immunological tolerance in general, as suggested by some results (Yunis *et al.*, 1974). A strong effect of hormonal status might also explain the different rates of appearance of mammary tumours in C3H and A.CA mice.

On the other hand, RIII and C3H milks have clearly different actions on the transplantability of C3H tumours. As the former is of European origin and the latter American, it may be expected that they are more different from each other than C3H-MTV and A.CA-MTV. Some antigenic specificities common to RIII-

MTV and C3H-MTV have been observed, however, using neutralizing antibodies raised in the rabbit (Blair *et al.*, 1970). Nevertheless, a difference in tolerance-inducing antigenic parts remains possible and would explain the results presented. On the other hand, the differential actions observed between RIII-MTV and C3H-MTV might also explain on a non-specific basis, the difference we observed. The immunosuppressive and/or immuno-stimulating action (Griswold *et al.*, 1973) of MTV seems to be a complex problem, and the effect could depend on both the mouse strain (Griswold *et al.*, 1973) and the MTV strain. Certainly, additional experiments are necessary to clarify this point.

We thank Dr G. Rudali and Mr L. Auseppe, Institut du Radium, Paris, for providing the RIII mice.

The technical help of Mrs M. C. Beugnot, Mr P. Mouchette and Mr A. Liegey is gratefully acknowledged.

This work was supported by the Institut National de la Santé et de la Recherche Médicale, Paris, Contract no. 74-5-004-02.

### REFERENCES

Barrett, M. K. & Morgan, W. C. (1949) Maternal Influence on Growth Rate of Transplantable Tumor in Hybrid Mice. *J. natn. Cancer Inst.*, **10**, 81.

Blair, P. B., Weiss, D. W. & Smith, G. H. (1970) Studies on Antigenic Cross-reactivity of Oncogenic RNA Viruses. Cross-reactions between Mouse Mammary Tumor Viruses from Different Mouse Strains. *Israel J. med. Sci.*, **6**, 611.

Blair, P. B., Kripke, M. L., Lappé, M. A., Bonhag, R. & Young, L. (1971) Immunologic Deficiency Associated with Mammary Tumor Virus (MTV) Infection in Mice: Hemagglutinin Response and Allograft Survival. *J. Immunol.*, **106**, 364.

Committee on Standardized Genetic Nomenclature for Mice (1960) Standardized Nomenclature for Inbred Strains of Mice, Second Listing. *Cancer Res.*, **20**, 145.

Griswold, D. E., Heppner, G. H. & Calabresi, P. (1973) Alteration of Immunocompetence by Mammary Tumor Virus. *J. natn. Cancer Inst.*, **50**, 1035.

Guggiari, M. & Rudali, G. (1977) Mammary Carcinogenesis in (C3H × RIII) F1 Mice under Different Experimental Conditions. *Biomedicine* **27**, 27.

Jerne, N. K. & Nordin, A. A. (1963) Plaque Formation in Agar by Single Antibody-producing Cells. *Science, N.Y.*, **140**, 405.

Morton, D. L. (1969) Acquired Immunological Tolerance and Carcinogenesis by the Tumour Virus. I. Influence of Neonatal Infection with the Mammary Tumor Virus on the Growth of Spontaneous Mammary Adenocarcinomas. *J. natn. Cancer Inst.*, **42**, 311.

Oth, D., Robert, F. & Dumont, F. (1972) Etudes Complémentaires sur " l'Effet Hybride " Présenté par des Tumeurs Mammaires Spontanées de la Souris C3H. p. 61. In " Recherches Fondamentales sur les Tumeurs Mammaires ", Ed. J. Mouriquand, Paris: INSERM.

Oth, D., Leclère, M., Grignon, G. & Burg, C. (1968) Maternal Factor in the " Hybrid Effect " Given by a Spontaneous Mammary Carcinoma of C3H Mice. *Life Sciences*, **7**, 599.

Sanford, B. H. & Soo, S. F. (1971) Resistance to Transplants of Recent Spontaneous Parental Line Tumors by F1 Hybrid Hosts. *J. natn. Cancer Inst.*, **46**, 95.

Vlahakis, G. (1973) Comparison of Three Mammary Tumor Viruses in Genetically Similar Susceptible Mice. *J. natn. Cancer Inst.*, **51**, 1711.

Yunis, E. J., Good, R. A., Smith, J. & Stutman, O. (1974) Protection of Lethally Irradiated Mice by Spleen Cells from Neonatally Thymectomized Mice. *Proc. natn. Acad. Sci. USA*, **71**, 2544.